# Enhanced polyhydroxybutyrate (PHB) production by newly isolated rare actinomycetes *Rhodococcus* sp. strain BSRT1-1 using response surface methodology

**DOI:** 10.1038/s41598-021-81386-2

**Published:** 2021-01-21

**Authors:** Chanaporn Trakunjae, Antika Boondaeng, Waraporn Apiwatanapiwat, Akihiko Kosugi, Takamitsu Arai, Kumar Sudesh, Pilanee Vaithanomsat

**Affiliations:** 1 Nanotechnology and Biotechnology Research Division, Kasetsart Agricultural and Agro-Industrial Product Improvement Institute (KAPI), Kasetsart University, Bangkok, Thailand; 2 Post-Harvest Science and Technology Division, Japan International Research Center for Agricultural Sciences (JIRCAS), Tsukuba, Ibaraki Japan; 3 School of Biological Sciences, Universiti Sains Malaysia, 11800 Gelugor, Penang Malaysia

**Keywords:** Biotechnology, Microbiology

## Abstract

Poly-β-hydroxybutyrate (PHB) is a biodegradable polymer, synthesized as carbon and energy reserve by bacteria and archaea. To the best of our knowledge, this is the first report on PHB production by a rare actinomycete species, *Rhodococcus pyridinivorans* BSRT1-1. Response surface methodology (RSM) employing central composite design, was applied to enhance PHB production in a flask scale. A maximum yield of 3.6 ± 0.5 g/L in biomass and 43.1 ± 0.5 wt% of dry cell weight (DCW) of PHB were obtained when using RSM optimized medium, which was improved the production of biomass and PHB content by 2.5 and 2.3-fold, respectively. The optimized medium was applied to upscale PHB production in a 10 L stirred-tank bioreactor, maximum biomass of 5.2 ± 0.5 g/L, and PHB content of 46.8 ± 2 wt% DCW were achieved. Furthermore, the FTIR and ^1^H NMR results confirmed the polymer as PHB. DSC and TGA analysis results revealed the melting, glass transition, and thermal decomposition temperature of 171.8, 4.03, and 288 °C, respectively. In conclusion, RSM can be a promising technique to improve PHB production by a newly isolated strain of *R. pyridinivorans* BSRT1-1 and the properties of produced PHB possessed similar properties compared to commercial PHB.

## Introduction

Petrochemical-derived plastics have many applications. Global economic growth and improvement in living standards has led to an increase in purchasing power, which has contributed to an increase in plastic production^1^. Although traditional petrochemical-derived plastic products have increased the quality of everyday life, they account for the accumulation of municipal waste, which persists undegraded for decades in the ecosystem^2^. Because of these challenges biodegradable plastics with lower or no negative impact on the environment have gained attention as replacements for petrochemical-derived plastics.

Polyhydroxyalkanoates (PHAs) is an intracellular storage compound accumulated as energy reserve by some microorganisms under stress^3,4^. PHA has thermo-mechanical properties similar to petrochemical polymers, such as polypropylene (PP) and polyethylene (PE)^5,6^. Based on their biodegradable, thermoplastic, and mechanical properties, PHAs are expected to replace petrochemical-derived plastics^7–9^. Among 150 PHA monomers^10^, poly-β-hydroxybutyrate (PHB), the most commonly synthesized form of PHA, has attracted more attention than others due to its physical, mechanical, and immunological properties, which make it an ideal candidate for applications in agriculture, food, and medicine^11,12^.

In addition to PHB producers, such as *Cupriavidus necator*, *Bacillus* sp*.*, *Pseudomonas* sp*.*, and *Escherichia coli* transformants^13–16^, certain actinomycetes also accumulate PHB granules. Studies on PHB production and degradation by *Streptomyces*, which is the dominant genus of actinomycete, have been reported^17,18^. However, only a few studies on PHB production by the rare actinomycete genus *Rhodococcus* have been conducted. Members of the *Rhodococcus* are widely distributed in nature; they have been isolated from soil, water, marine sediments, and other sources^19^. They belong to the non-sporulating and mycolic acid-rich group within actinomycetes, together with other related genera, including *Mycobacterium*, *Nocardia*, *Corynebacterium*, and *Gordonia*^20^. *Rhodococcus* is an excellent candidate for bioremediation and bioconversion because it can significantly degrade and transform a wide variety of natural organic and xenobiotic compounds via diverse catabolic pathways^21^. Additionally, Members of *Rhodococcus*, such as *R. aetherivorans*^21^, *R. ruber*^22^, *R. equi*^23^, and *R.* jostii^24^, produce PHAs using various carbon sources, including sugars, oils, hydrocarbons, and agricultural waste. However, *R. pyridinivorans*, which was isolated in this study, has not been reported to be a PHA producer.

Optimization of fermentation medium is critically investigated because it plays a critical role in cell growth and expression of preferred metabolite affecting overall productivity^25^. It should be carried out before large-scale metabolite production. Various non-statistical and statistical techniques for medium optimization have been studied extensively. The non-statistical, one-factor-at-a-time (OFAT) approach is identifies significant parameters and their effective ranges. However, OFAT requires numerous experiments to explain the effect of individual parameters and is time consuming. Moreover, it rarely evaluates the effect of more than one factor and its interactions at a time, which is a disadvantage once the interactions of parameters are significant^26^. Thus, statistical experimental design methods are required to provide statistical models, which investigate several independent variables simultaneously and characterize the relationship between the variables^27^. Response surface methodology (RSM) is a statistical optimization method, which employs experimental factorial designs, such as central composite design (CCD), for optimizing process yield and defines the behavior of the response in the selected design space^28,29^. CCD is used to study the interaction effect of the factors that significantly affect product formation. The experimental runs of CCD work as inputs for RSM in finding the mathematical model that links process parameters and outcome^30^.

The aim of this study was to isolate and identify PHB-producing bacteria from the soil, optimize the fermentation medium components by using RSM to enhance PHB production as well as PHB characterization, and to improve the cellular biomass of PHB-producing bacteria in a 10 L stirred-tank bioreactor.

## Results

### Isolation and screening of PHB-producing bacteria

A total of 79 bacterial strains were successfully isolated from the wastewater treatment area of Kasetsart University, Bangkok, Thailand. Nile red agar plates were used for preliminary screening to select PHB-producing strains. Ten strains exhibited bright orange fluorescence under UV light after being incubated on MM agar containing 1% (w/v) glucose supplemented with Nile red for 3 days (data not shown). However, BSRT1-1 accumulated the highest amount of PHB, at 18 wt% DCW, when cultured in PHB production medium. BSRT1-1 colonies were opaque and raised, with regular configuration. BSRT1-1 produced orange colonies when grown on NA and TSA agar plates at room temperature (35 °C). Microscopic examination revealed that BSRT1-1 cells were Gram-positive, non-spore-forming, and non-motile with a rod–coccus morphology. Cells were short rods during the exponential growth phase and converted to cocci during the stationary growth phase.

### Identification of PHB-producing bacteria by 16S rRNA gene

**Figure 1:**
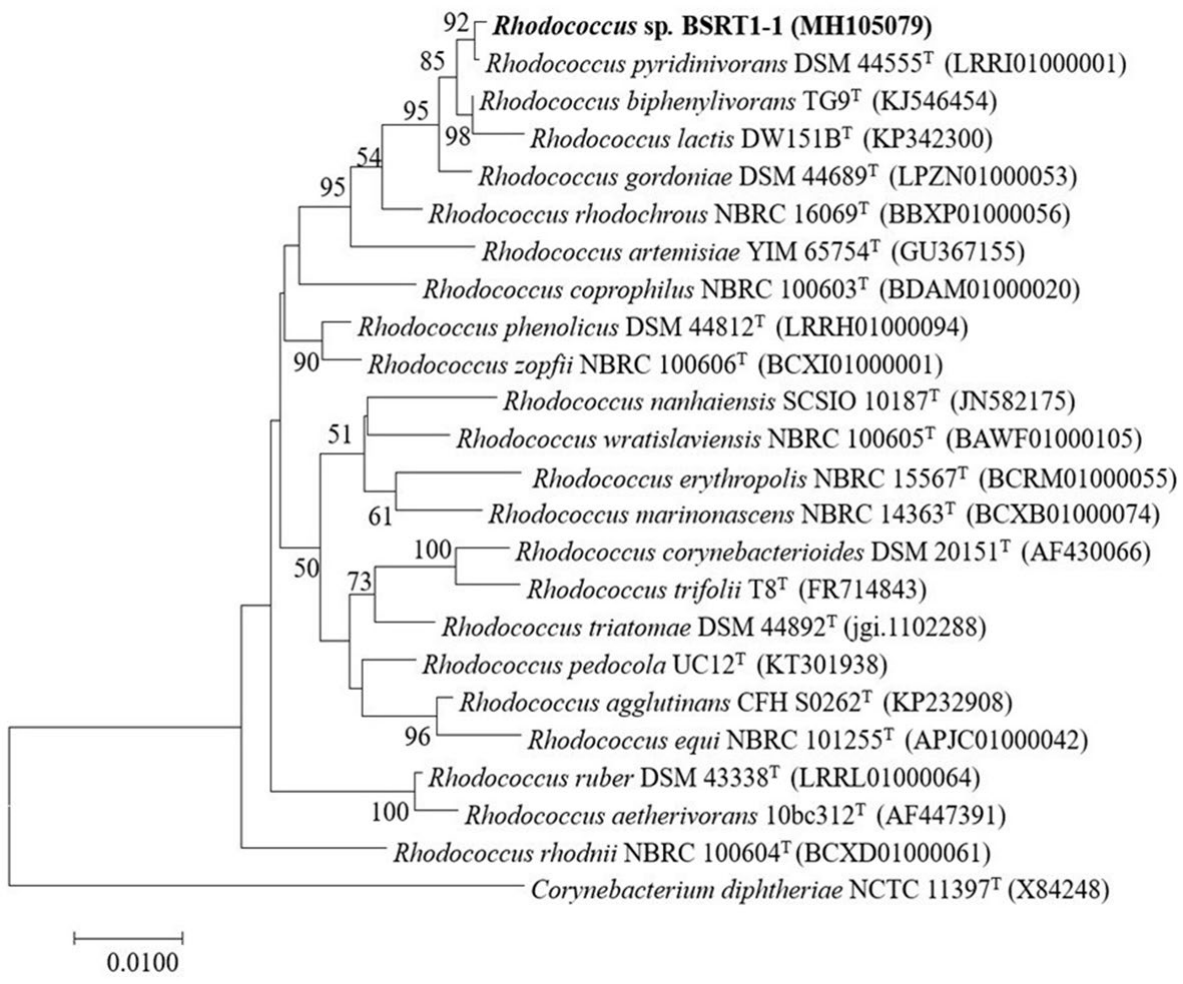
Neighbor-joining tree, based on 16S rRNA gene sequences, showing the position of BSRT1-1 and closely related species of *Rhodococcus*. Numbers at nodes indicate levels of bootstrap support (%) based on neighbor-joining analysis of 1000 resampled datasets; only values ≥ 50% are given. Filled circles indicate branches of the tree that were also recovered using the maximum-parsimony and maximum-likelihood tree-making algorithms. *Corynebacterium diphtheriae* NCTC 11397 T (GenBank Accession No. X84248) was used as an outgroup. Bar, 0.01 substitutions per site.

To identify BSRT1-1, the 16S rRNA gene of strain BSRT1-1 was extracted and sequenced. The sequence of the 16S rRNA gene (1,483 bp) was obtained and used for the initial BLAST search. Blast analysis of 16S rRNA gene sequence of BSRT1-1 revealed significant similarity with that of *R. pyridinivorans* DSM44555^T^ (99.86%), *R. biphenylivorans* TG9^T^ (98.45%), *R. gordoniae* DSM 44689^T^ (99.17%), and *R. lactis* DW151B^T^ (98.81%). To determine the taxonomic position of BSRT1-1, a phylogenetic analysis was performed to compare its 16S rRNA gene sequence with that of other species of *Rhodococcus*. The strain BSRT1-1 formed a coherent clade with *R. pyridinivorans* DSM44555^T^ in the NJ phylogenetic tree reconstructed using 16S rRNA gene sequences from various strains of *Rhodococcus*. BSRT1-1 also formed a cluster with the type strains of *R. pyridinivorans* (Fig. 1). *Rhodococcus* species, such as *R. aetherivorans*^20^ and *R. equi*^23^, produce PHB.

### Selection of carbon and nitrogen source

**Figure 2:**
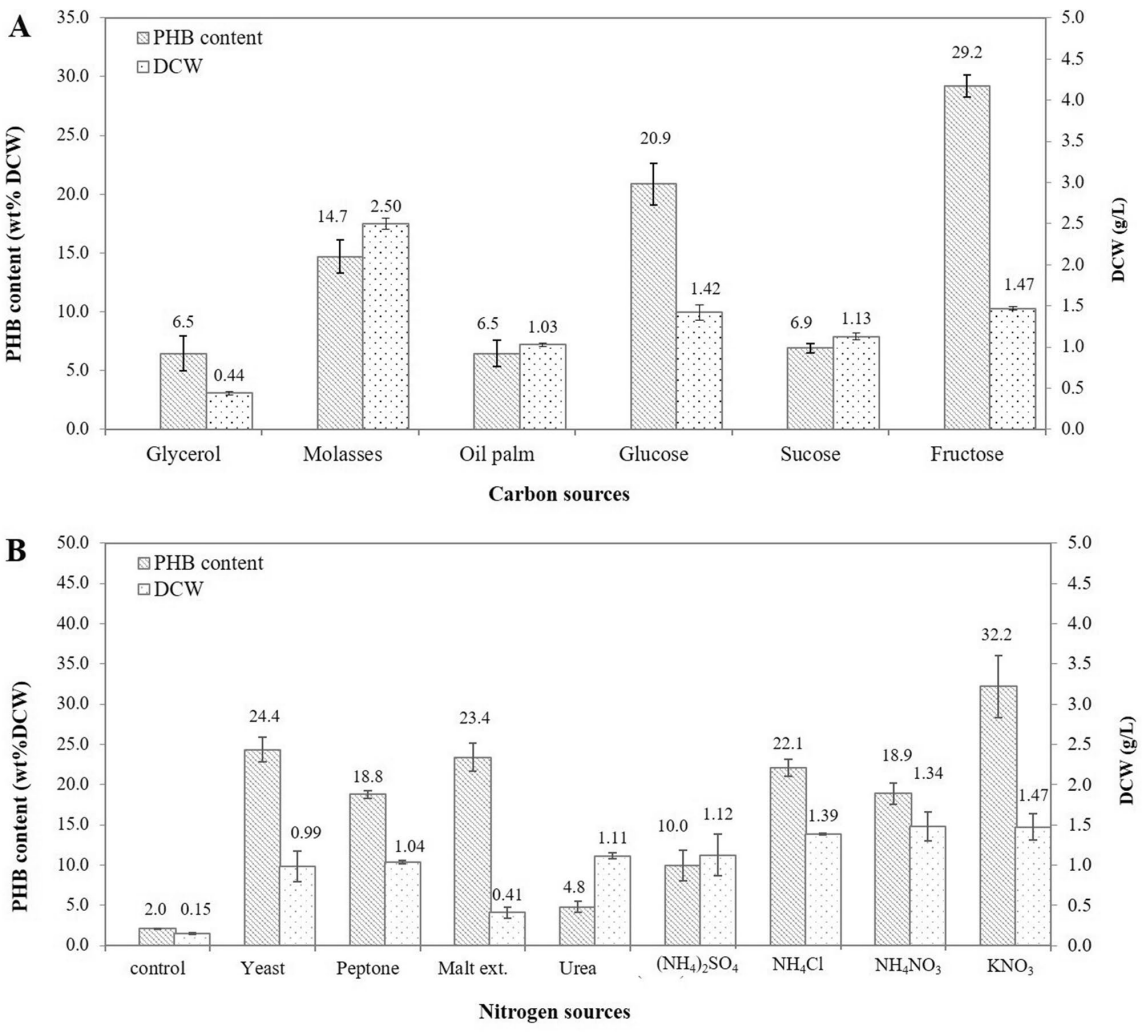
Poly-β-hydroxybutyrate (PHB) production by *Rhodococcus pyridinivorans* BRST1-1 using different carbon and nitrogen sources in shake flask experiments.

PHA biosynthesis was performed in a 250-mL flask to evaluate PHB production in *R. pyridinivorans* BSRT1-1 and to select the best carbon and nitrogen source for further optimization studies. BSRT1-1 was cultured under nitrogen-limiting conditions using various carbon and nitrogen sources. Of six carbon sources, i.e., glucose, fructose, sucrose, glycerol, molasses, and oil palm, fructose was found to be the best carbon source for PHB production. Therefore, fructose was selected as the carbon source for optimization experiments. BSRT1-1 could grow and accumulate up to 22 wt% DCW PHB when using 30 and 0.5 g/L of fructose and NH_4_Cl as carbon and nitrogen source, respectively (Fig. 2A). Approximately 1–2.5 g/L of DCW and 6–22 wt% DCW of PHB content were achieved using glucose, fructose, sucrose, molasses, and oil palm as a carbon source, whereas only 0.4 g/L of DCW was obtained when using glycerol as a carbon source. Thus, in addition to simple sugars (monosaccharides), BSRT1-1 could use other carbon sources, such as molasses and oil palm, for cell growth and PHB production (Fig. 2A).

Nitrogen source is also an important parameter for PHB accumulation. The effects of various nitrogen sources, such as yeast extract, malt extract, peptone, urea, (NH_4_)_2_SO_4_, NH_4_Cl, NH_4_NO_3_, and KNO_3_, on cell growth and PHB production by BSRT1-1 were tested (Fig. 2B). A maximum biomass and PHB content of 1.47 g/L and 32.2 ± 4 wt% DCW were obtained when 0.5 g/L of potassium nitrate (KNO_3_) and 30 g/L of fructose were used as a nitrogen source and carbon source, respectively. Therefore, KNO_3_ was used as a nitrogen source for optimization experiments.

### Optimization of PHB accumulation by RSM

A three-variable-five-level design of CCD was used to determine the optimized medium composition for PHB accumulation and the interactive effects of each parameter. Fructose, KNO_3_, and TE solution were selected as the parameters for CCD. The response data were analyzed by the Design-Expert v7.0.0 software (Stat-Ease, Inc. MN, USA). The experimental results of PHB content and predicted responses are shown in Table 1. The results indicated that the highest PHB content, 42.9 wt% DCW, was obtained when the concentrations of fructose, KNO_3_, and TE solution were 20, 1.0, g/L, and 1.0 mL/L, respectively. The lowest PHB content was 17.4 wt% DCW, when the concentrations of fructose, KNO_3,_ and TE solution were 3.20, 1.0, g/L, and 1.0 mL/L, respectively. The results obtained from multiple regression analyses of CCD experiments were fitted to a second-order polynomial model. PHB content fitted in terms of coded variables was obtained as the following model:

$$ \begin{aligned} {\mathrm{Y}} & = 42.67 - 0.36{\mathrm{X}}1 - 2.55{\mathrm{X}}2 - 3.28{\mathrm{X}}3 - 8.42{\mathrm{X}}1{\mathrm{X}}2 + 0.95{\mathrm{X}}1{\mathrm{X}}3 - 2.21{\mathrm{X}}2{\mathrm{X}}3 \\ & \quad - 6.65{\mathrm{X}}1^{2} - 4.02{\mathrm{X}}2^{2} - 2.91{\mathrm{X}}3^{2} \\ \end{aligned} $$

where Y is the PHB content and X1, X2, and X3 are coded values of fructose, KNO_3_, and TE solution, respectively. The statistical significance of the equation was verified by the F test and the ANOVA for the response surface quadratic model is shown in Table S1. The regression equation presented a determination coefficient, R^2^ = 0.9011 (Table S1). Thus, this model can explain approximately 90.11% of the variability in the dependent variable; 9.89% was affected by other variables. The R^2^ value is always between 0 and 1. The closer the R^2^ to 1.0, the stronger the model and the better it predicts the response^31^. The adjusted R^2^, which corrects the R^2^ value for the sample size and the number of terms, was 0.7739^32^.

**Table 1:** Experimental design and result of central composite design (CCD) of response surface methodology.

Run no	Level			PHB content (%DCW)		Dry cell weight (g/L)	PHB concentration (g/L)
	X1	X2	X3	Observed	Predicted		
1	− 1	− 1	− 1	24.7	25.6	2.1 ± 0.0	0.5 ± 0.01
2	1	− 1	− 1	40.5	39.8	2.1 ± 0.1	0.9 ± 0.01
3	− 1	1	− 1	28.5	41.7	2.0 ± 0.0	0.6 ± 0.01
4	1	1	− 1	38.5	22.3	2.2 ± 0.0	0.8 ± 0.02
5	− 1	− 1	1	33.7	21.6	2.1 ± 0.1	0.7 ± 0.01
6	1	− 1	1	21.2	39.6	1.9 ± 0.2	0.4 ± 0.01
7	− 1	1	1	21.8	28.9	1.3 ± 0.3	0.3 ± 0.03
8	1	1	1	40.8	13.2	2.2 ± 0.2	0.9 ± 0.02
9	− 1.68	0	0	17.4	24.5	1.9 ± 0.1	0.3 ± 0.04
10	1.68	0	0	23.5	23.3	1.2 ± 0.2	0.3 ± 0.01
11	0	− 1.68	0	34.0	35.6	3.1 ± 0.1	1.1 ± 0.02
12	0	1.68	0	40.9	27.0	2.2 ± 0.1	0.9 ± 0.02
13	0	0	− 1.68	41.2	40.0	2.5 ± 0.2	1.0 ± 0.02
14	0	0	1.68	38.7	28.9	2.3 ± 0.1	0.9 ± 0.01
15	0	0	0	42.7	42.7	2.9 ± 0.0	1.2 ± 0.04
16	0	0	0	42.9	42.7	3.0 ± 0.2	1.3 ± 0.05
17	0	0	0	42.5	42.7	3.1 ± 0.3	1.3 ± 0.01

The P-values are used to check the significance of each coefficient, which help to understand the pattern of mutual interactions between the best variables^33^. The smaller the P-value, the larger the significance of the corresponding coefficient^34^. The F test and the corresponding P-values were estimated, as shown in Table 2. The model indicates that the constant linear (X3), quadratic (X1^2^, X2^2^), and interaction terms (X1X2 and X2X3) are significant (p < 0.05) (Table 2). In this model, the negative polynomial coefficient in interaction terms implies that the interaction is antagonistic. Quadratic model analysis shows that the input independent variable of TE solution (X3) was important for PHB accumulation. However, the quadratic terms coded as X12, X22 and their interaction (X1X2) are also significant, with the probability value of p < 0.05, which indicates that the effect of coded variable X1, X2 and their interactions are considerable for PHB accumulation.

**Table 2:** Analysis of variance table.

Source	Sum of squares	Degree of freedom	Mean square	F value	p-value Prob > F
Model	1404.8	9	156.09	7.09	0.0086*
X_1_	1.7385	1	1.7385	0.08	0.7869
X_2_	89.084	1	89.084	4.04	0.0843
X_3_	147.18	1	147.18	6.68	0.0362*
X_1_X_2_	566.86	1	566.86	25.73	0.0014*
X_1_X_3_	7.1683	1	7.1683	0.33	0.5862
X_2_X_3_	39.087	1	39.087	1.77	0.2246*
X_1_^2^	498.47	1	498.47	22.63	0.0021*
X_2_^2^	182.29	1	182.29	8.27	0.0238*
X_3_^2^	95.668	1	95.668	4.34	0.0757

R^2^ = 0.9011, Adj-R^2^ = 0.7739.

*Statistically significant at 95% probability level.

**Figure 3:**
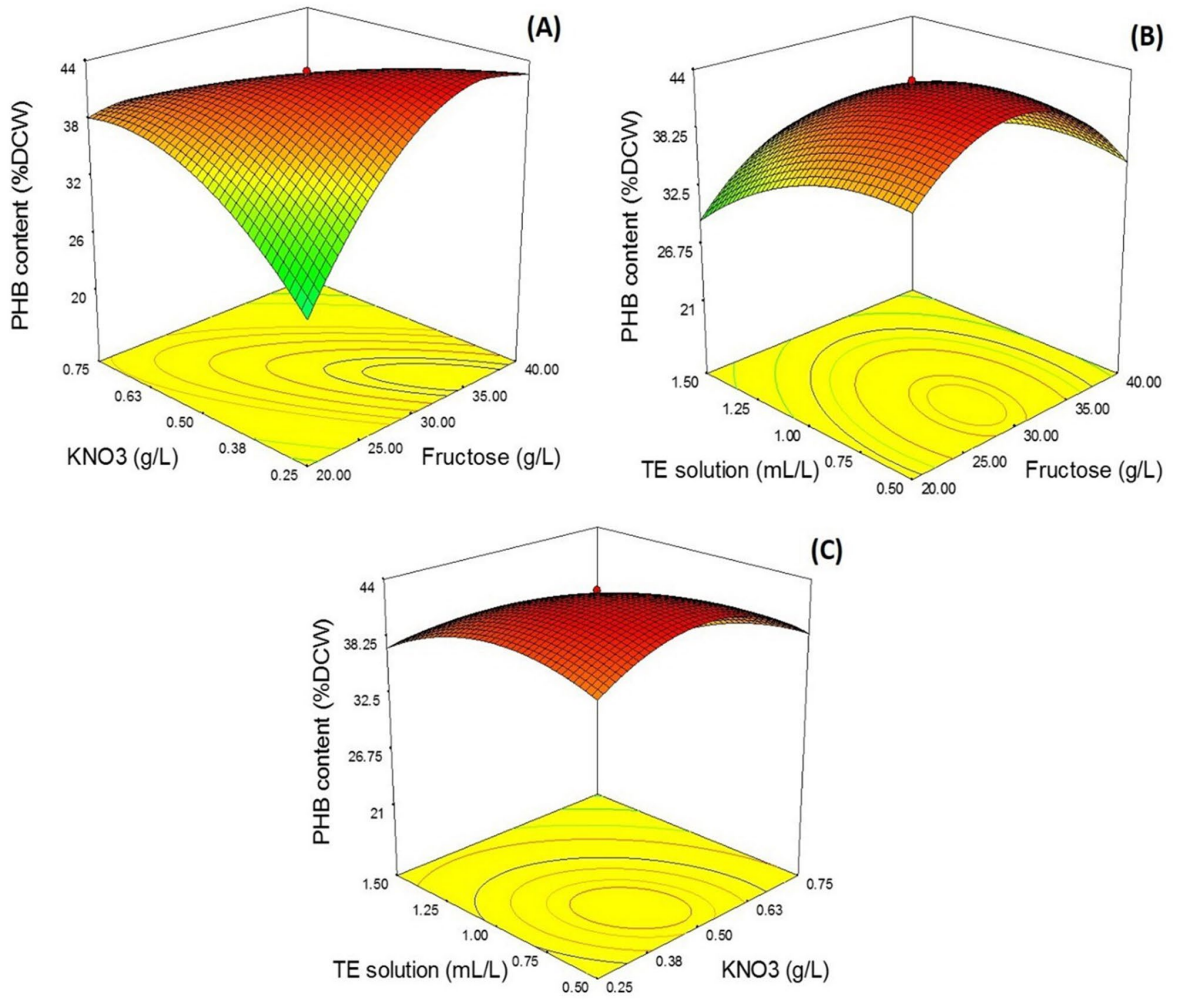
Response surface and contour plots described by the model, representing poly-β-hydroxybutyrate (PHB) accumulation (wt% DCW) as a value of fructose, KNO_3_, and TE solution by *Rhodococcus pyridinivorans* BRST1-1. Combined effect of fructose and KNO_3_ (**A**); fructose and TE solution (**B**); KNO_3_ and, TE solution (**C**).

To evaluate the interaction between different parameters and to determine the optimal concentration of each parameter for maximum PHB content, the response between fructose (X1), KNO_3_ (X2), and TE solution (X3) was plotted, as shown in the Fig. 3. Figure 3A shows the effect of fructose and KNO_3_ on PHB content. PHB content increased when fructose concentration increased from 30.0 to 35.0 g/L. At a higher fructose concentration (> 35.0 g/L), PHB content declined. PHB content increased with decreasing KNO_3_ concentration, from 0.5 to 0.3 g/L. At a high KNO_3_ concentration (> 0.3 g/L) PHB content declined. The effect of fructose and TE solution on PHB content is shown in Fig. 3B. PHB content increased with decreasing fructose, from 30.0 to 29.0 g/L. PHB content declined at a higher concentration of fructose (> 29.0 g/L), whereas PHB content increased with an increase in TE concentration, from 0.5 to 0.6 mL/L. PHB content declined at a higher concentration of TE solution (> 0.6 mL/L). The effect of KNO_3_ and TE solution are shown in Fig. 3C. PHB content increased with decreased KNO_3,_ from 0.50 to 0.45 g/L. PHB content decreased at a higher concentration of KNO_3_ (> 0.45 g/L) and increased with increased concentration of TE solution, from 0.5 to 0.75 mL/L. PHB content declined when TE solution was at > 1.0 mL/L.

The model was validated for the three variables within the design space to confirm the optimization results. Optimized medium composition from RSM was carried out in a 250-flask scale in triplicate. The result shows that under the following conditions: fructose, 33.6 g/L, KNO_3_, 0.3 g/L, and 1.0 mL/L of TE solution, the maximum PHB content of 43.1 wt% DCW, with 3.2 g/L of DCW, nearing the predicted PHB content of 43.36 wt% DCW. The predicted values and actual experimental values were compared and the residual was calculated. The percentage error between the actual and predicted values for PHB content was 0.31%. Hence, the observed models were reasonably accurate and RSM analysis is a suitable technique for predicting and optimizing the fermentation media.

### Scaling up PHB production in a 10 L bioreactor

To enhance the biomass and PHB accumulation of *R. pyridinivorans* BRST1-1, batch cultivation was carried out in a 10 L stirred-tank bioreactor containing 6 L of optimized media (fructose, 33.6 g/L, KNO_3_, 0.3 g/L, and 1.0 mL/L of TE solution). The temperature, pH, aeration rate, and agitation speed were fixed at 35 °C, 7.0, 0.75 vvm, and 180 rpm, respectively. During 72 h of fermentation, growth of BSRT1-1 showed a predictable exponential phase, followed by PHB accumulation. The quantity of PHB accumulated increased in the fermenter as the fructose levels decreased (Fig. S1). As seen in Fig. S1, the biomass increased gradually over the fermentation period. However, when the fermentation period was extended above the optimum (54 h), with no remaining fructose, PHB accumulation and cell growth were interrupted and the degradation of PHB began^35^. The highest production of PHB was at 48 h when the DCW was 5.2 ± 0.5 g/L; PHB content was 46.8 ± 2 wt% DCW (Fig. S1).

### Characterization of PHB

**Figure 4:**
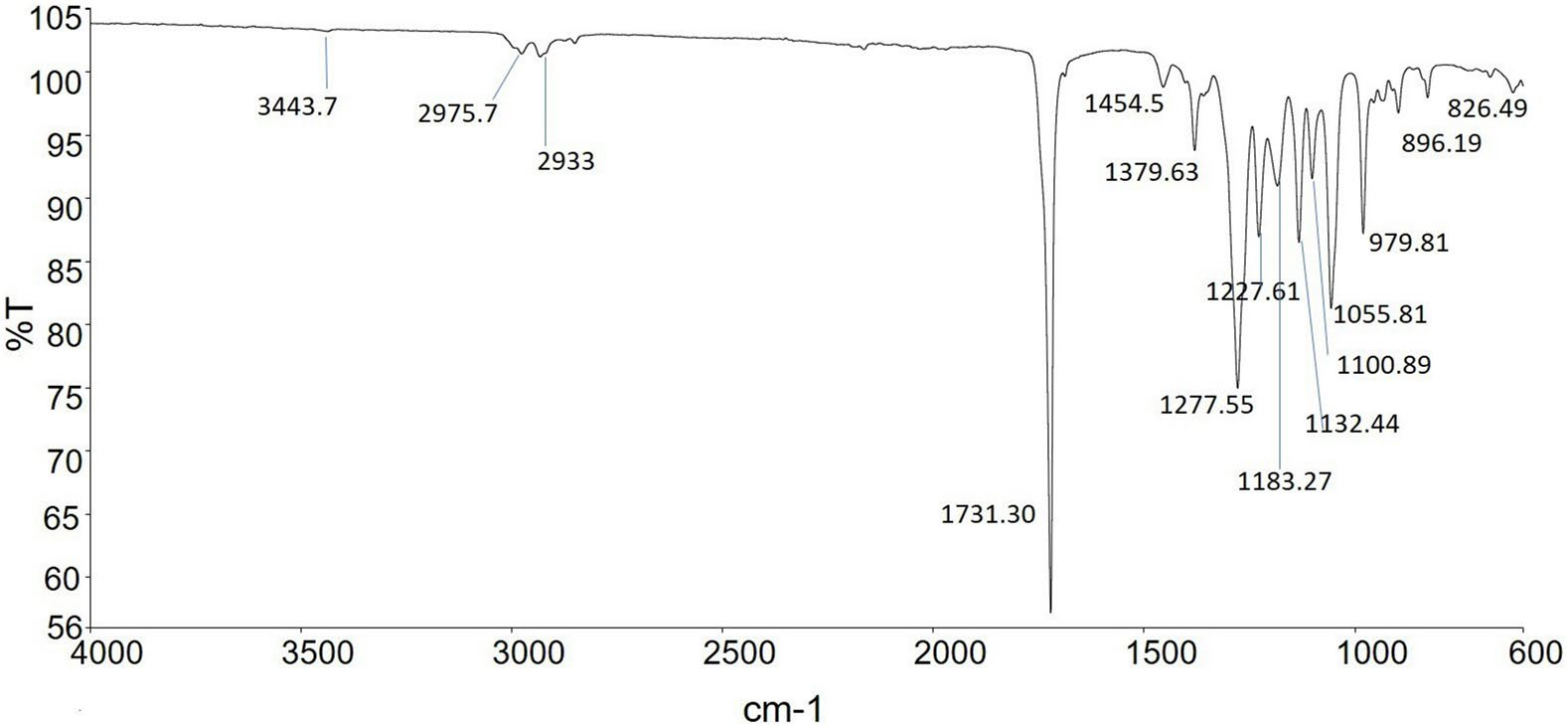
Fourier transform infrared spectroscopy (FTIR) spectrum of PHB produced by *Rhodococcus pyridinivorans* BRST1-1.

**Figure 5:**
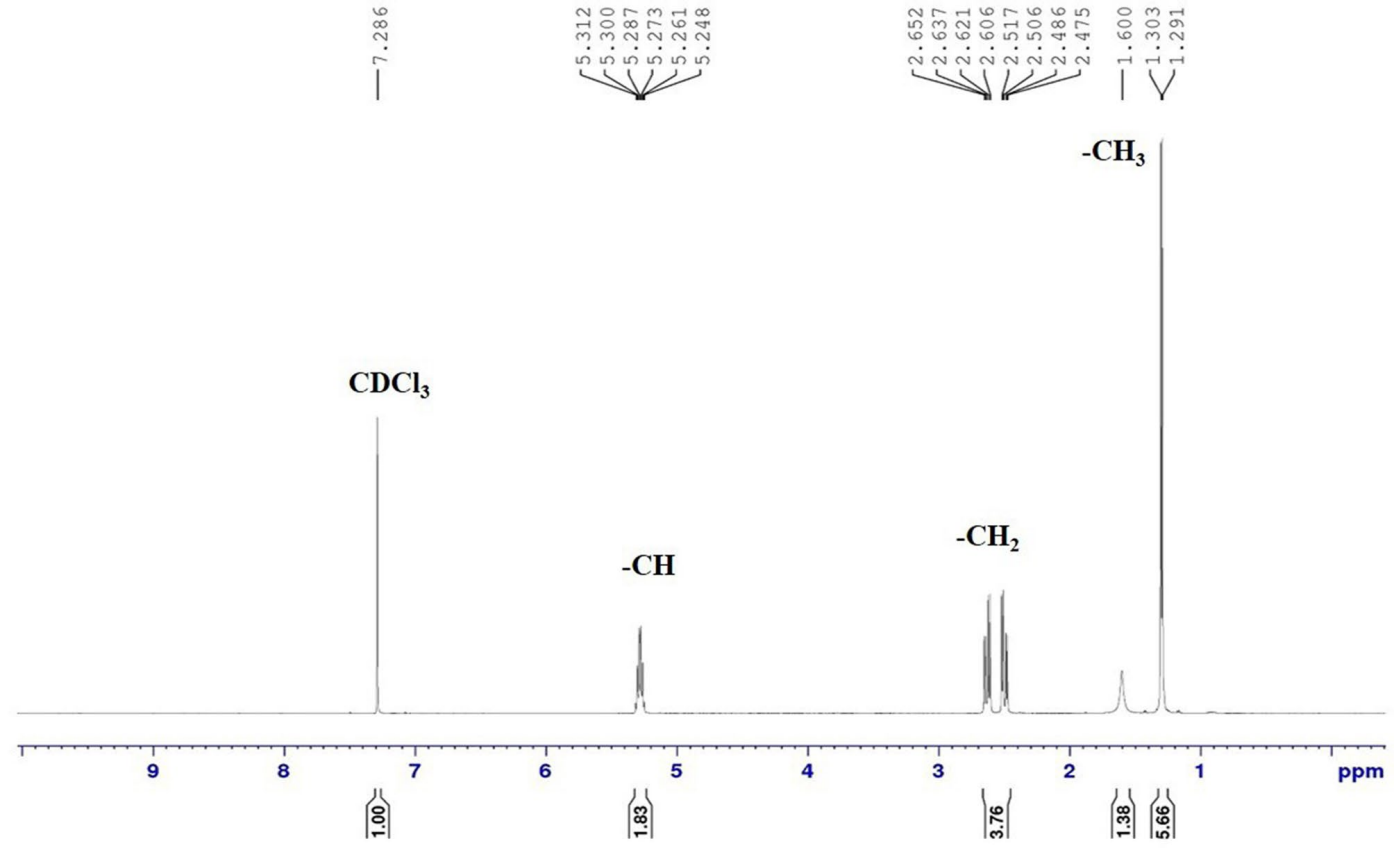
Proton Nuclear Magnetic Resonance Spectroscopy (^1^H NMR) spectrum of PHB produced by *Rhodococcus pyridinivorans* BRST1-1.

**Figure 6:**
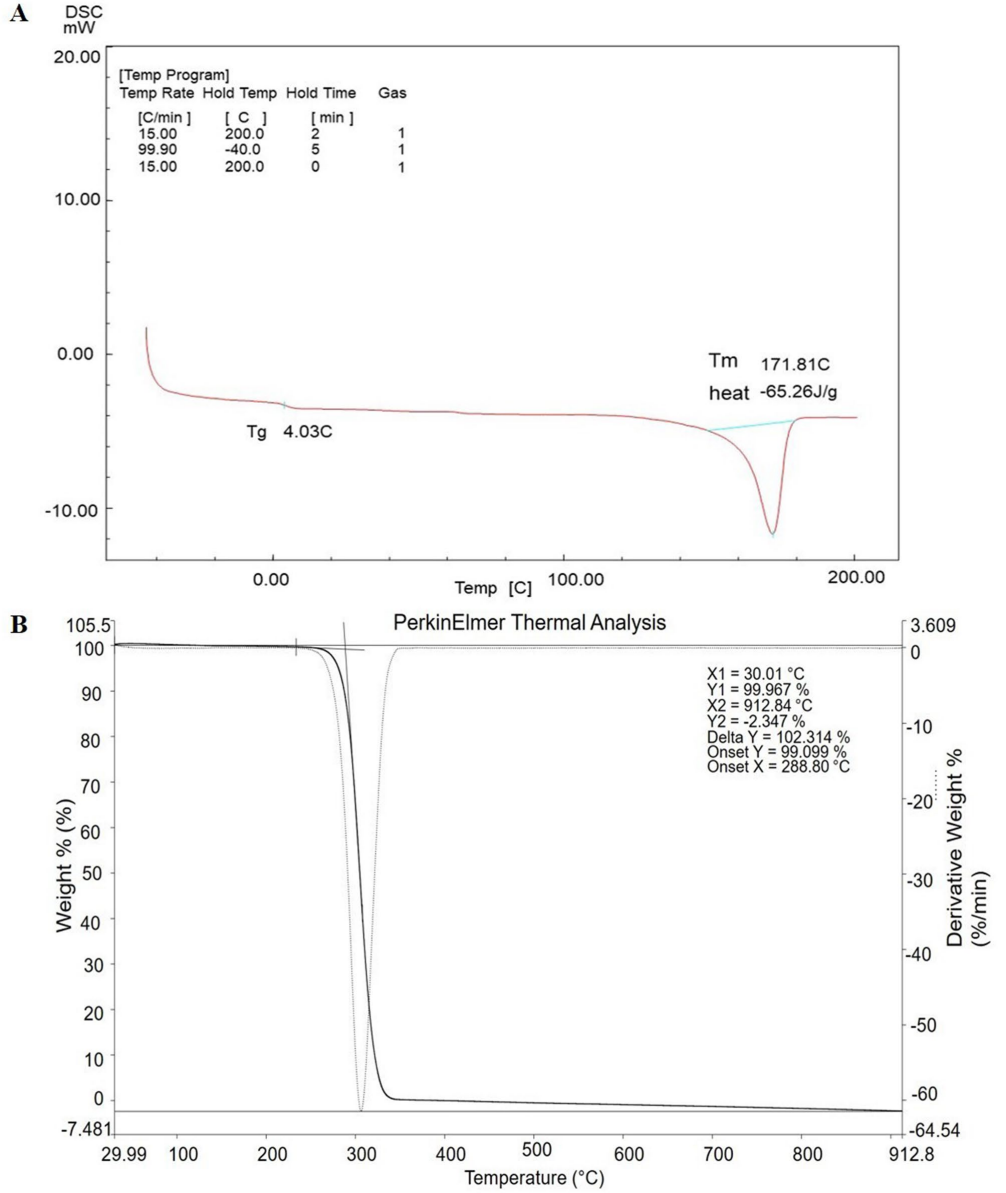
Differential Scanning Calorimetry (DSC) and Thermogravimetric analysis (TGA) of PHB produced by *Rhodococcus pyridinivorans* BRST1-1.

Fourier Transform IR spectroscopy (FTIR) was performed to investigate the different functional groups of PHB produced by *R. pyridinivorans* BSRT1-1. The FTIR spectrum of PHB, which was recorded between 4000 and 600 cm^−1^ (Fig. 4), shows a sharp absorption band at 1721 cm^−1^ which corresponds to carbonyl (C=O) stretching of the ester and another band at 1277 cm^−1^, corresponding to the -CH group. The presence of these bands has been reported and labeled as a PHB marker^36^. While a series of bands between 1,000 and 1,300 cm^−1^ show stretching of the C–O bond of the ester group^37^. The bands at 2975 and 2933 cm^−1^ indicate the presence of methyl (CH_3_) and methylene (CH_2_) asymmetric and symmetric stretching modes, respectively. Additionally, bands of minor relevance at 3443.7 cm^−1^ are related to a terminal OH group^38^. The ^1^H NMR was performed to observe the chemical structure of PHB synthesized by the strain BSRT1-1. Figure 5 shows the ^1^H NMR spectrum of three different signals at 1.29, 2.5, and 5.27 ppm, which were represented methyl, methylene, and methane group, respectively, confirming its structure as a PHB^39,40^. Thermal properties of PHB synthesized by strain BSRT1-1 was performed by using DSC and TGA analysis (Fig. 6). DSC was conducted to investigate the melting temperature (*T*_m_) and glass transition (*T*_g_) of PHB. The *T*_m_ and *T*_g_ of PHB were found to be 171.8 and 4.03 °C, respectively (Fig. 6A). TGA was performed to observe the thermal stability of PHB synthesized by strain BSRT1-1. Figure 6B shows the PHB degradation pattern, which was exhibited a single degradation step under a nitrogen atmosphere, between 240 °C and 400 °C. The result indicates that PHB degradation appears rapidly, marked by a sharp decrease in the curve. The onset temperature of the PHB was at 288 °C. The PHB was completely degraded at 320 °C.

## Discussion

PHB is a currently well-studied type of PHAs, which is an intracellular storage compound accumulated as energy reserve material by bacteria and archaea under different stress conditions^3,4^. In the present study, potential PHB accumulating bacteria were successfully isolated from the wastewater treatment area of Kasetsart University, Bangkok, Thailand. Preliminary screening of PHB-producing strains was further identified by a Nile red agar plates method^41^. This method has been used commonly for the rapid identification of PHA-producing bacteria, but not yet with archaea^42^. Among all PHB-producing isolated strain, the strain BSRT1-1 was found to be the best PHB producer, which was accumulated the highest amount of PHB, at 18 wt% DCW, when cultured in PHB production medium using glucose and NH_4_Cl as a carbon and nitrogen source, respectively. The present study shows that the habitats of the wastewater treatment area were a potential source for bacterial isolates producing PHB. Many studies have been reported on the isolation of PHA-producing bacteria from wastewater treatment sources. Yan et al.^43^ have been isolated PHA-accumulating bacteria from activated sludge samples collected from municipal wastewater treatment plants in Quebec by using acetate as sole carbon source. Besides, Bhuwal et al.^44^ reported the use of pulp, paper, cardboard industry sludge, and wastewater for the isolation and screening of PHA accumulating bacteria. Additionally, Jinda and Paniticharoenwong^45^ have been successfully isolated PHA-producing bacteria, *Ralstonia* sp. NBKT10 frm the soil of palm oil manufacturing plants.

Comparison of the bacterial 16S rRNA gene sequence has emerged as a preferred molecular technique to the identification of bacteria that has replaced the conventional techniques that rely on phenotypic identification^46^. In this study, the most excellent PHB producer strain, BSRT1-1, was identified based on the 16S rRNA gene as *Rhodococcus pyridinivorans*. The *R. pyridinivorans* was first isolated as a pyridine-degrading coryneform bacterium from industrial wastewater in Korea^47^. This species has been reported the ability to degrades various type of aromatic compounds, for example, pyridine^47^, styrene^48^, as well as BTX (benzene, toluene, and xylene)^49^. However, interestingly, this is the first report on PHB production by *R. pyridinivorans* strain BSRT1-1.

The effect of various carbon and nitrogen sources on PHB production was investigated by using OFAT method. *R. pyridinivorans* BSRT1-1 could grow and accumulate the maximum PHB of 32.19 ± 3.86 wt% DCW when using 30 and 0.5 g/L of fructose and potassium nitrate as carbon and nitrogen source, respectively. Therefore, fructose and potassium nitrate were used for optimization experiments. This finding is supported by the previous report, where fructose has been reported as a suitable substrate for PHB production in *Alcaligenes eutrophus*^50^. Similarly, *Aquitalea* sp. USM4 can accumulate up to 27 wt% of PHA when 10 g/L of sugars such as glucose, fructose, and sucrose are used as a carbon source^51^. In comparison, toluene and crude palm kernel oil are used as a carbon source for PHA production by *R. aetherivorans*^20^ and *R. equi*^22^, respectively. Both organic and inorganic nitrogen sources were attempted to enhance the nitrogen source for PHB production. In this study, potassium nitrate, an inorganic nitrogen source, supported to produce the highest amount of PHB. Contrary to this, urea has been reported as a suitable nitrogen source for PHB production by *Aquitalea* sp. USM4^51^ and *Pseudomonas aeruginosa*^52^. While the highest level of PHB accumulation by *Bacillus subtilis* 25 and *Bacillus megaterium* 12 was observed in a medium using an organic nitrogen source, protease peptone^53^.

RSM employing CCD was applied to improve the production of PHB in a flask scale. The highest yield of 3.60 ± 0.5 g/L in biomass and 43.1 ± 0.5 wt% of dry cell weight (DCW) of PHB were achieved when using RSM optimized medium, which was increased the production of biomass and PHB content by 2.5 and 2.3-fold, respectively. Previously, RSM has been reported as a powerful tool to improve the production of PHB by various microorganisms. Higher concentrations of PHB can be produced from glucose by a newly engineered strain of *C. necator* NSDG-GG using RSM^26^. PHB production by *Methylobacterium sp* has been successfully enhanced by RSM using methanol as a sole carbon source^54^. RSM is useful in improving PHB production by the *B. drentensis* strain BP17 using pineapple peel as a sole carbon source^55^. Hassan et al.^56^ have been reported the efficient optimization of PHB production by novel *Bacillus subtilis* from rice bran using RSM employing Box–Behnken design. Moreover, RSM enhances the production of PHA copolymers, such as poly(3-hydroxybutyrate-*co*-3-hydroxyvalerate) (PHBV). The development of PHBV production using sugarcane molasses supplemented with the co-substrates palm oil and corn steep liquor as a carbon source is reported for the yeast strain, *Wickerhamomyces anomalus* VIT-NN01^57^. Besides, RSM has been used to evaluate the optimum operating condition for PHBV-tapioca starch composites^58^. However, when comparing PHB production by *R. piridinivorans* BSRT1-1 with *C. necator*, which is industrially important strain for PHB production, under nutrient limitation with an excess of carbon, *C. necator* accumulated PHA (mainly PHB) up to 90% of its DCW^59^, whereas *R. piridinivorans* BSRT1-1 can accumulate 43% PHB of its DCW when using RSM optimized medium. Nevertheless, the enhancement of biomass to improve PHB production by *R. piridinivorans* BSRT1-1 can be achieved by scaling up PHB production in a 10 L bioreactor.

The PHB production in a 10 L stirred-tank bioreactor can improve the production of biomass by 1.4. Thus, significantly higher biomass could be obtained in a larger scale bioreactor. The improvement of PHB production using batch fermentation by various types of bacteria has been reported^60–62^. However, in this present study PHB accumulation cannot be increased by this approach due to the disadvantages of single batch fermentation^35^. Batch fermentations are the most popular and straightforward method for PHB production, however inherently low yield. The maximum permitted concentration of nutrients is limited by the physiological requirements of the processing strain at the beginning of the fermentation batch^2^.

The extracted PHB was characterized by FTIR, NMR, DSC and TGA techniques. The observed band in the FTIR spectrum at 1721 cm^−1^, 1277, 1000–1300, 2975, 2933, and 3443.7 cm^-1^ represented C=O ester, -CH, C–O, CH_3_, CH_2,_ and OH groups of the polymer, respectively. The obtained FTIR analysis is similar to the previous reports^36–39^. Additionally, three different signals of ^1^H NMR spectrum at 1.21, 2.56, and 5.22 ppm represented methyl, methylene, and methane groups, respectively, which were confirmed the chemical structure of the PHB^40^. Thermal analyses showed that the extracted PHB existed as a thermally stable semi-crystalline polymer^55^, the *T*_m_ and *T*_g_ of extracted PHB were 171.8 and 4.03 °C, respectively. Similar *T*_m_ and *T*_g_ have been previously reported in PHB^63–65^. The maximum thermal decomposition observed was at 288 °C by FIIR and is related with the ester cleavage of PHB by b-elimination reaction^67^. Many researchers have been reported similar TGA results of PHB^55,63–66^. All these results confirmed that the polymer produced by *R. pyridinivorans* BSRT1-1 is PHB homopolymer, and the properties of extracted PHB were similar to the commercial PHB^68^.

## Conclusions

The strain *R. pyridinivorans* BSRT1-1 was isolated from soil and identified as the first PHB producer in *R. pyridinivorans*. Fructose and KNO_3_ were found to be the best carbon and nitrogen sources for PHB production by this strain, respectively. Under optimum conditions, obtained from RSM, this strain can accumulate 43.1 wt% DCW of PHB and produce 3.60 ± 0.5 g/L of biomass. The optimized medium can improve the production of biomass and PHB content by 2.5 and 2.3-fold when compared to un-optimized medium. Therefore, RSM is a powerful tool for optimizing PHB production. Furthermore, higher biomass of 5.2 ± 0.5 g/L and PHB content of 46.8 ± 2 wt% DCW were achieved from the 10 L stirred-tank bioreactor. Finally, the functional group and chemical structure results verified the polymer as PHB and the thermal properties of produced PHB possessed similar properties compared to commercial PHB.

## Materials and methods

### Sample collection

A total of 12 soil samples were randomly collected from the wastewater treatment area of Kasetsart University, Bangkok, Thailand (latitude: 13.854529N, longitude: 100.570012 E). All soil samples were kept in sterilized envelopes and brought to the laboratory. Each sample was air-dried at room temperature (35 °C) for 1–2 days, crushed, and mixed.

### Medium and inoculum preparation

The minimal medium (MM) for PHB production consisted of NH_4_Cl, 0.5 g/L; KH_2_PO_4_, 2.8 g/L; Na_2_HPO_4_, 3.32 g/L; MgSO_4_·7H_2_O, 0.25 g/L, and 1 mL/L of trace element (TE) solution. The TE solution comprised: ZnSO_4_·7H_2_O, 1.3 g/L; FeSO_4_·7H_2_O, 0.2 g/L; (NH_4_)_6_Mo_7_O_24_·4H_2_O, 0.6 g/L; H_3_BO_3_, 0.6 g/L, and CaCl_2_, 0.2 g/L. The sugars were sterilized at 110 °C for 20 min and then aseptically added into the flask containing other components. The pH of the final culture medium was adjusted to 7.0 before bacterial inoculation. The inoculum of the selected strain was prepared by inoculating a full loop of a single colony in a 250 mL Erlenmeyer flask containing 50 mL of Tryptic Soy Broth (TSB) (BD, Franklin Lakes, NJ, USA). The culture incubated at 35 °C with shaking at 180 rpm for 24 h. The cells were harvested by centrifugation at 8,000 g at 4 °C for 10 min. The cell pellet was washed with sterile 0.85% (w/v) NaCl. The optical density of cell suspension was adjusted using 0.85% (w/v) NaCl to 0.5–07 at 600 nm. A 10% (v/v) of cell suspension was used as the inoculum.

### Isolation and screening of PHB-producing bacteria

One gram of each soil sample was serially diluted in sterile distilled water and plated onto nutrient agar (NA) and TSA plates. All plates were incubated at 37 °C for 3 days. Several individual colonies of different morphologies were picked and the purified isolates were maintained on agar slants of the same medium. All the isolated strains were streaked onto mineral medium (MM) agar plates containing glucose, 30 g/L; Nile red, 0.005% (w/v) (Sigma-Aldrich, St. Louis, MO, USA), and 15 g/L of agar powder to screen for PHB production. The plates were incubated at 37 °C for 1–3 days. Thereafter, colonies with bright orange fluorescence under UV were selected. The isolates were stored at -80 °C in 20% (v/v) glycerol until further use.

### Identification of PHB-producing bacteria by 16S rRNA gene

The selected PHB-producing isolate was identified based on 16S rRNA sequence. The DNA was extracted using the standard protocol of Sambrook and Russell (2001)^69^. 16S rRNA gene amplification was carried out using Ex Taq polymerase (TakaRa Bio Inc., Tokyo, Japan). A forward primer (27F): 5′AGA GTTTGATCCTGGCTAG 3′ and reverse primer (1492R): 5′GGCTA CCTTGTTACG ACTT 3′ were used to amplify the gene. The PCR temperature cycling conditions were as follows: initial denaturation at 94 °C for 5 min; 30 cycles of denaturation at 94 °C for 1 min, annealing at 55 °C for 1 min, and elongation at 72 °C for 2 min. The final cycle was followed by extension at 72 °C for 10 min^70^. The amplification products were purified using the Qiagen PCR purification kit (Qiagen) and subcloned to *p*TAC-1, followed by transformation into *E. coli* JM109. Plasmids were extracted with the QIAprep Spin Miniprep kit (Qiagen) and sequenced by MACROGEN (Korea). The GeneBank database in the BLAST program of the National Center for Biotechnology Information was used to compare the sequence of 16S rRNA gene, which was deposited in GenBank. The phylogenetic tree was constructed using the MEGA software version 7.0.

### Selection of carbon and nitrogen source

OFAT method was used to investigate the effect of carbon and nitrogen source on PHB production by the selected strain. Briefly, MM medium supplemented with 30 g/L of six carbon sources, i.e., glucose, fructose, sucrose, glycerol, molasses, and oil palm, was inoculated with 10% (v/v) of inoculum and the cultures were grown at 35 °C with shaking at 180 rpm for 72 h. Thereafter, the samples were analyzed and the best carbon source for PHB production was selected and used for nitrogen source studies. To evaluate the effect of the nitrogen source on PHB production, eight nitrogen sources, i.e., yeast extract, malt extract, peptone, urea, (NH_4_)_2_SO_4_, NH_4_Cl, NH_4_NO_3_, and KNO_3_, were used at a concentration of 0.5 g/L. All experiments were performed in triplicates and average values were determined.

### Experimental design and statistical modeling

In this experiment, CCD was used to design fermentation experiments. RSM, which is an empirical modeling technique, was applied to evaluate the relationship between a set of controllable experimental factors and observed results. The Design-Expert v7.0.0 software (Stat-Ease, Inc. MN, USA) was used for statistical DOE and the data was analyzed. According to this design, the total number of treatment combinations was 2^*k*^ + 2*k* + *n*_0_, where *k* is the number of independent variables and *n*_0_ is the number of repetitions of experiments at the center point^71^. Seventeen fermentation runs were designed based on the CCD of three factors—fructose concentration, X1 (g/L); KNO_3_ concentration, X2 (g/L); and TE solution volume, X3 (mL/L). Each variable was coded at five levels (− 1.68, − 1, 0, + 1, and + 1.68) to describe the nature of the response surface in the optimum region. The coded and actual levels of the variables are shown in Table 3. The design matrix of the performed fermentation runs is shown in Table 1. The average values were reported from duplicate experimental runs. The coded values were set for three factors, resulting in seven factorial points (including all possible combinations of the maximum and minimum levels), seven axial points (one of the factors set at the midpoint), and three center points (replicated fermentation runs at the factors midpoint). The experimental results of CCD design were fit with a second-order polynomial equation by a multiple regression technique, as shown in Eq. (1).

1 $$ Y = \beta_{0} + \sum\limits_{i = 1}^{k} {\beta_{i} X_{i} } + \sum\limits_{i = 1}^{k} {\beta_{ii} X_{i}^{2} } + \sum\limits_{i < } {\sum\limits_{j = 2}^{k} {\beta_{li} X_{i} X_{j} } } $$

where *Y* is the predictive measured response; *X*_i_ and *X*_j_ are the independent variables; *β*_0_ represents the intercept; and *β*_i_, *β*_ii_, and *β*_lj_ are the regression coefficients of the model^72^. The generated model for three independent variables is shown in Eq. (2).

2 $$ \begin{aligned} Y & = \beta_{0} + \beta_{1} X_{1} + \beta_{2} X_{2} + \beta_{3} \beta_{3} + \beta_{11} X_{1}^{2} + \beta_{22} X_{2}^{2} + \beta_{33} X_{3}^{2} \\ & \quad + \beta_{12} X_{1} X_{2} + \beta_{13} X_{1} X_{3} + \beta_{23} X_{2} X_{3} \\ \end{aligned} $$

where *Y* is the predictive measured response as PHB content (wt% Dry cell weight (DCW)); *β*1, *β*2, and *β*3 are linear coefficients; *β*11, *β*22, and *β*33 denote quadratic coefficients; *β*12, *β*13, and *β*23 are interaction coefficients; X1, X2, and X3 represent coded values of fructose concentration, X1 (g/L); KNO3 concentration, X2 (g/L); and TE solution volume, X3 (mL/L).

**Table 3:** Experimental code and actual levels.

Independent variables	Unit	Range and levels				
		− 1.68	− 1.00	0.00	+ 1.00	+ 1.68
Carbon source, X1	g/L	3.20	10.0	20.0	30.0	36.8
KNO_3_, X2	g/L	0.16	0.5	1.0	1.5	1.84
TE solution, X3	mL/L	0.16	0.5	1.0	1.5	1.84

### Model validation and confirmation

To determine the accuracy of the model, the concentrations of three factors (fructose, KNO_3_, and TE solution), which had a significant influence on PHB production, were randomly selected within the design space to confirm the shake flask model by *R. pyridinivorans* BRST1-1*.* The remaining components of the medium in this experiment were at fixed levels.

### Scale up in the 10 L bioreactor

Fermentation was evaluated in a 10 L stirred-tank bioreactor (Model MDFT-N-10L, Marubishi, Japan) to enhance the production of biomass and PHB by *R. pyridinivoran*s BRST1-1. The inoculum was prepared in a 500 mL Erlenmeyer flask containing 200 mL of media. Batch cultivation was carried out at 35 °C in a 10 L stirred-tank bioreactor containing 6 L of optimized media. The bioreactor was sterilized in an autoclave at 121 °C for 30 min, cooled, and then inoculated with 10% (v/v) inoculum. The pH of the culture broth was maintained at pH 7.0 by the addition of acid or base by a pH controller. The airflow rate and agitation speed were fixed at 0.75 vvm and 180 rpm, respectively. The cell biomass and PHB content were evaluated every 6 h for 72 h of fermentation. The fermentation experiments were carried out in duplicates and average values were determined.

### Dry cell weight (DCW) analysis

For the determination of DCW, 1 mL of cell culture suspension was added in triplicate to pre-weighed Eppendorf tubes. The cells were harvested by centrifugation at 8,000 rpm at 4 °C for 10 min. Thereafter, the harvested cells were washed twice by resuspending the cell pellet in distilled water and centrifuged again at 8,000 rpm at 4 °C for 10 min. The washed cell pellet was frozen at − 20 °C overnight. Subsequently, the cell pellet was lyophilized using a freeze-dryer for 2 days. Eppendorf tubes were weighed again to confirm stability and the DCW was calculated in g/L.

### PHB content analysis

The PHB content was measured as described by Karr et al.^73^ Briefly, 50 mL of stationary growth phase culture was collected by centrifugation at 8,000 rpm at 4 °C for 10 min. The harvested cells were washed twice with distilled water and frozen overnight at − 20 °C. The dry pellets were boiled in 1 mL concentrated H_2_SO_4_ for 60 min, diluted with 4 mL of 0.014 M H_2_SO_4_, and filtered through an MCE filter. Samples were analyzed for PHB concentration by high-performance liquid chromatography using an Aminex HPX -87H ion-exclusion column. Crotonic acid (Sigma-Aldrich) was used as a standard. The regression equation obtained from the crotonic acid standard was used to calculate the amount of crotonic acid produced from PHB.

### PHB extraction and purification

The PHB accumulated in the cells were extracted using chloroform extraction method which was modified by Hassan et al.^74^ Briefly, the PHB was extracted by dissolving 1 g of freeze-dried cells in 100 mL chloroform for 3–5 days at room temperature. After that, the solution was filtered using Whatman No. 1 filter paper to remove the cell debris. The filtrate was concentrated to 10 mL using a rotary evaporator followed by drop wise addition into a vigorously stirred 100 mL of chilled methanol. The purified polymer was finally collected, and air dried for 3 days.

### Fourier transform IR spectroscopy (FTIR)

The functional groups of purified PHB were identified by ATR-FTIR spectrophotometer equipped with spectrum (analysis software) for Windows v.10 (PerkinElmer, USA). The following conditions were used: Spectral range, 4000–600 cm^−1^; window material, CsI; 16 scans; resolution 4 cm^−1^.

### Proton nuclear magnetic resonance spectroscopy (^1^H NMR)

The chemical structure of PHB was confirmed by proton nuclear magnetic resonance (^1^H-NMR) spectroscopy. Around 3 mg of the purified PHB was dissolved in 1 mL of deuterated chloroform (CDCl_3_) at a concentration of 25 mg/mL using tetramethysilane as an internal chemical shift reference. The ^1^H-NMR spectra were recorded at 500 MHz on a Bruker AVANCE 500 (NC, USA) spectrometer at 30 °C.

### Differential scanning calorimetry (DSC) analysis

DSC experiments was performed using DSC-60 (Shimadzu, Japan) instrument under a nitrogen flow rate of 30 mL/min. Approximately 5 mg of purified PHB was loaded into an aluminum pan and heated from 25 to 200 °C at a heating rate of 15 °C/min. The melt samples were then maintained at 200 °C for 2 min and followed by rapid quenching to -40 °C. They were heated again from -40 to 200 °C at a heating rate of 15 °C/min. The melting temperature (T_*m*_) and glass transition temperature (T_*g*_) were determined from DSC thermogram.

### Thermogravimetric analysis (TGA)

The thermal degradation temperature of the PHB was analyzed by TGA using instrument STA 6000 (Perkin Elmer, USA). About 5 mg of the purified PHB sample was loaded in aluminum pan and heated from 30 to 920 °C at a heating rate of 20°C/min under nitrogen atmosphere.

## Supplementary Information

Supplementary Figure S1.

Supplementary Table S1.
