# Supplementary figures and images for: Enhanced polyhydroxybutyrate (PHB) production by newly isolated rare actinomycetes Rhodococcus sp. strain BSRT1-1 using response surface methodology
Source: Sci Rep. 2021 Jan 21;11:1896. doi: 10.1038/s41598-021-81386-2 (PMC7820505; doi:10.1038/s41598-021-81386-2)

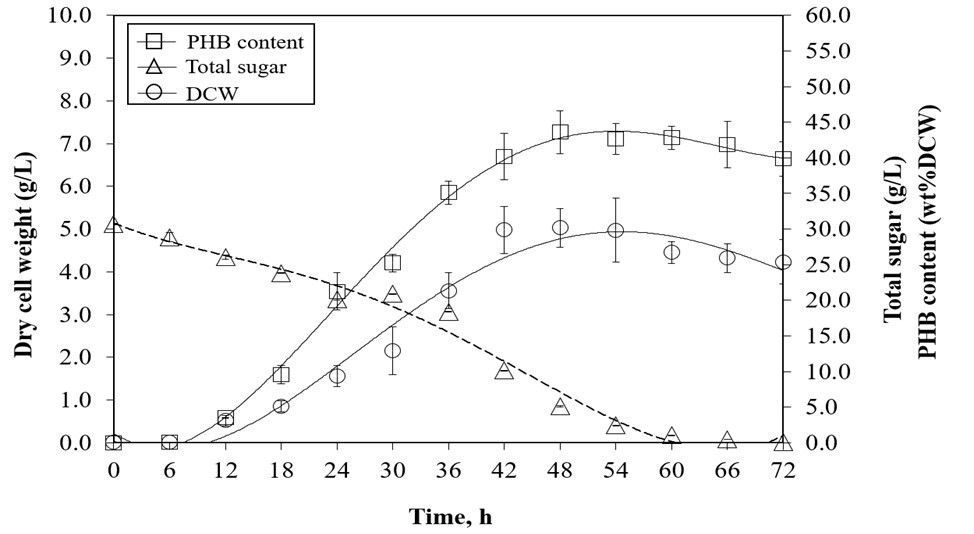


**Figure S1** The PHB productionof *R. pyridinivorans* BSRT1-1 in 10L stirred-tank bioreactor.

Supplement: Supplementary file 1 — Supplementary Figure S1. [file 41598_2021_81386_MOESM1_ESM.docx]
