# Supplementary material for: Enhanced polyhydroxybutyrate (PHB) production by newly isolated rare actinomycetes Rhodococcus sp. strain BSRT1-1 using response surface methodology
Source: Sci Rep. 2021 Jan 21;11:1896. doi: 10.1038/s41598-021-81386-2 (PMC7820505; doi:10.1038/s41598-021-81386-2)
Supplement: Supplementary file 2 — Supplementary Table S1. [file 41598_2021_81386_MOESM2_ESM.docx]

**Table 1** Analysis of variance (ANOVA) for the model regression representing PHB content.

| **Source** | **Sum of squares** | **Degree of freedom** | **Mean squares** | **F-value** | **P-value** |
| --- | --- | --- | --- | --- | --- |
| **PHB content** |  |  |  |  |  |
| Model | 1404.84 | 9 | 156.09 | 7.09 | 0.0086 |
| Residual | 154.22 | 7 | 22.03 |  |  |
| Lack of fit | 153.92 | 5 | 30.78 | 211.36 | 0.0047 |
| Total | 1559.05 | 16 |  |  |  |
| R^2^ = 0.9011 |  |  |  |  |  |
| Adj-R^2^ = 0.7739 |  |  |  |  |  |
|  |  |  |  |  |  |
